# Sex Differences in the Efficacy of Mediterranean Diet Treatment: A Nutrigenomics Pilot Study

**DOI:** 10.3390/genes14111980

**Published:** 2023-10-24

**Authors:** Laura Di Renzo, Paola Gualtieri, Giulia Frank, Gemma Lou De Santis, Rossella Cianci, Giulia Bigioni, Antonino De Lorenzo

**Affiliations:** 1 Section of Clinical Nutrition and Nutrigenomics, Department of Biomedicine and Prevention, University of Rome Tor Vergata, Via Montpellier 1, 00133 Rome, Italy; laura.di.renzo@uniroma2.it (L.D.R.); paola.gualtieri@uniroma2.it (P.G.);; 2 PhD School of Applied Medical-Surgical Sciences, University of Rome Tor Vergata, Via Montpellier 1, 00133 Rome, Italy; gemmaloudesantis@gmail.com; 3 School of Specialization in Food Science, University of Tor Vergata, Via Montpellier 1, 00133 Rome, Italy; 4 Department of Translational Medicine and Surgery, Catholic University of the Sacred Heart, Fondazione Policlinico Universitario A. Gemelli, IRCCS, 00168 Rome, Italy; 5 Department of Physics, University of Rome Sapienza, 00185 Rome, Italy

**Keywords:** Mediterranean diet, nutrigenomics, gene expression, sex difference

## Abstract

The Mediterranean diet (MedD) has been shown to have beneficial effects on health, well-being, and mental status. It potentially modulates gene expressions linked to oxidative stress, contributing to its beneficial effects on overall health. The aim of this study was to assess the effects of MedD treatment in healthy human volunteers on the expression of ten genes related to oxidative stress and inflammation in women and men. Of 30 enrolled subjects, 17 were eligible, 10 women and 7 men. All of them received the same MedD treatment. Before and after 8 weeks of MedD treatment, an evaluation of body composition, blood tests, and anthropometric and clinical parameters was performed. Furthermore, 10 genes were amplified and analyzed. The study showed significant differences between females and males in body composition and biochemical parameters before and after MedD treatment. Significant differences between females and males in Resistance Force (*p* < 0.009) and Diastolic Blood Pressure (*p* < 0.04) before MedD treatment, and in High-Density Lipoprotein (*p* < 0.02) after MedD treatment, were observed. Moreover, a significant upregulation of *Apolipoprotein E* and *Angiotensin I-Converting Enzyme* in females has been shown. Sex differences impact MedD treatment response, and influence the genetic expression of genes related to oxidative stress; our findings may help to personalize diet therapy and contribute to overall health and well-being.

## 1. Introduction

Several factors, such as lifestyle, diet, environmental burden, and genetics, significantly regulate individual health outcomes.

Oxidative stress occurs when there is a failure in neutralizing the production of reactive oxygen species (ROS) by using antioxidants. Oxidative stress can contribute to several disorders, including type 2 diabetes, cardiovascular disease (CVD), neurodegenerative and gastrointestinal disorders, and cancer [1].

Oxidative stress can disrupt cellular homeostasis, triggering inflammation and influencing gene expression, which orchestrates responses to the resulting cellular damage.

Dysregulation of oxidative stress and pro-inflammatory genes’ expression can significantly affect health and disease [2]. The intricate interplay between inflammation and oxidative stress underscores the importance of maintaining a delicate balance to ensure proper cellular function and prevent the onset and the progression of various diseases.

Adding antioxidants to diet, such as vitamins and plant-derived phenolic compounds, could support a favorable balance between pro-oxidative and antioxidative factors [3].

The Mediterranean diet (MedD) has been shown to potentially modulate gene expression related to oxidative stress, contributing to its beneficial effects on overall health [4]. The MedD is characterized by an abundance of fresh fruits and vegetables, whole grains, olive oil, lean proteins such as fish and poultry, and a moderate intake of dairy products, particularly yogurt and cheese. Additionally, it emphasizes the use of flavorful herbs and spices like basil, oregano, and garlic. Nuts and seeds, like almonds and sunflower seeds, are also included.

Particularly, it was observed that the consumption of 25 g of extra virgin olive oil successfully influenced the antioxidant profile and the expression of genes associated with inflammation and oxidative stress, including *Superoxide Dismutase 1* (*SOD1*), *Catalase* (*CAT*), and *Upstream Transcription Factor 1* (*USF-1*), during the post-meal period. Furthermore, a robust decrease in triglycerides, oxidized low-density lipoprotein (ox-LDL), the Visceral Adiposity Index (VAI), and malondialdehyde was observed [5].

Additionally, it was noted that a daily intake of 40 g of hazelnuts led to a decrease in post-meal risk factors associated with atherosclerosis, including ox-LDL, and also impacted the expression of genes linked to inflammation and oxidative stress [6].

Sex hormones, such as estrogens, regulate adipogenesis and metabolism. Estrogens present genomic and non-genomic effects; estrogen receptors mediate transcriptional activity and epigenetic changes, such as DNA methylation and demethylation, and histone and chromatin modifications with subsequent regulation of gene expression.

For example, the estrogens and their receptors have an impact on adipocyte growth and function, leading to enhanced expression of genes responsible for reducing ROS through antioxidants [7].

Understanding the influence of sex on gene expressions could potentially lead to the development of targeted nutrigenomics interventions to mitigate the negative effects of inflammation and oxidative stress-related pathways.

This study investigates the response to the MedD by examining potential differences between male and female participants, in order to gain a deeper understanding of how sex-related factors may modulate the effectiveness of antioxidants in mitigating inflammation and oxidative stress. Therefore, we set up a pilot clinical trial involving healthy human volunteers to explore the impact of MedD treatment on the expression of ten genes associated with oxidative stress and inflammation in females and males.

## 2. Materials and Methods

### 2.1. Subjects

Thirty healthy subjects gave informed consent to participate in the clinical pilot study. The healthy subjects were consecutively recruited within a program of routine medical check-ups at the Section of Clinical Nutrition and Nutrigenomics at the University of Rome, Tor Vergata. All study participants provided their consent for participation by reading and signing the informed consent form, according to the Helsinki Declaration of 1975, as revised in 2013. The trial is registered under NCT01890070 and approved by the Ethics Committee of the Calabria Region Central Area Section (register protocol no. 146 17 May 2018).

To be eligible for the study, participants met the following inclusion criteria: males and females, aged between 18 and 65 years, body mass index (BMI) > 19 kg/m^2^. Exclusion criteria were: pregnancy, active smoking, high blood pressure (≥140/90 mmHg), BMI > 40 kg/m^2^, acute illnesses, autoimmune and intestinal disorders, HIV/AIDS, neoplasms, vegetarianism, and use of vitamin supplements or antioxidants or any medication that could have an impact on inflammation and oxidative stress. All participants were in good health and exhibited no signs of chronic illness.

Participants were advised to maintain their regular physical activity levels and lifestyle throughout the study. Any instances of illness or abnormalities arising during the study period were required to be reported.

Patients were subjected to biweekly telephone interviews, during which they provided a nutritional recall of their dietary intake over the past 24 h. This served to assess their adherence to the dietary regimen and allowed for inquiries about any encountered complications or adverse effects. After each treatment period, a professional doctor evaluated the potential adverse effects of the interventions. This evaluation involved reviewing a checklist of symptoms, such as bloating, fullness, altered bowel habits, dizziness, and other potentially intervention-related symptoms. All participants successfully completed the study. No changes to trial outcomes after the trial commenced occurred.

The minimal number of subjects to enroll in the pilot study has been defined according to previous nutrigenomic studies [8].

### 2.2. Dietary Assessment and Mediterranean Diet Treatment

Both at the baseline and during the MedD treatment, a food frequency questionnaire was used to identify the weekly frequency of intake of different foods [9].

All the enrolled subjects received the same MedD treatment, personalized according to each patient’s energy requirements, derived by the De Lorenzo formula from TBLean [10].

The mean distribution of calories across meals was as follows: 15% for breakfast, 10% for the morning snack, 35% for lunch, 10% for the afternoon snack, and 30% for dinner.

Regarding the daily macronutrient intake of the MedD, it was categorized as follows: 40–45% of the total daily caloric intake from carbohydrates, 25–30% from proteins (with over 50% of these sourced from vegetables), 25–30% from lipids (with specific criteria: polyunsaturated fatty acids (PUFA) at 6–10%, a n-6/n-3 PUFA ratio of 3:1, monounsaturated fatty acids (MUFA) at 15%, and trans-fatty acids at <1%), saturated fat <10%, along with a fiber intake of 25 g.

MedD treatment was characterized by the following key features: seasonal consumption of fresh vegetables and fruits; inclusion of cereals and legumes; utilization of foods abundant in monounsaturated and polyunsaturated fats, such as olive oil, nuts, avocado, fatty fish, and low-fat fresh cheeses, while limiting saturated fats; preference for lean, organically sourced meat; and reliance on herbs and spices to minimize the use of added salt. This dietary pattern excludes processed and preserved foods, including frozen pre-packaged meals, cured meats, canned products, and sausages, as well as sugars, sugary alcoholic, and soft beverages.

The Mediterranean Adequacy Index (MAI) was calculated by assessing an individual’s adherence to the MedD based on specific dietary components commonly found in this dietary pattern. The MAI was calculated by assessing the percentage of daily caloric intake (% kcal/day) obtained from traditional Mediterranean foods like vegetables, fruits, fish, extra virgin olive oil, pasta, bread, and wine, in relation to non-traditional foods such as eggs, meat, dairy products, milk, sugar, sweets, and alcoholic beverages. A higher MAI score reflects a stronger adherence to the MedD, indicating a dietary pattern that includes a greater proportion of health-promoting foods. A value MAI of 5 is considered acceptable and 100% adequate if >15 [11].

### 2.3. Evaluation of Body Composition

Anthropometric and body composition data were evaluated before and after eight weeks (8-wks) of MedD treatment. Weight, height, and waist circumference were measured according to standard procedures [12].

Body mass index (BMI) was calculated as body mass (kg)/height (m^2^), and classified as recommended by Pujia et al. [13].

The waist-to-height ratio (W/H) was also calculated using a 0.5 cutoff point [14].

Resistance, reactance, impedance, and phase angle at 50 kHz frequency were assessed using a BIA phase sensitive system (BIA 101S, Akern/RJL Systems, Florence, Italy), according to standard procedures [15]. To determinate total body water (TBW) (%), the formula proposed by De Lorenzo et al. [16] was used.

Body composition analysis was conducted by DXA (Primus, X-ray densitometer; software version 1.2.2, Osteosys Co., Ltd., Guro-gu, Seoul, Republic of Korea) at baseline, according to the previously described procedure, to assess soft tissues, total body fat (TBFat), total body lean (TBLean), bone tissue, and total body bone (TBB) [17].

To calculate the percentage of total body fat (PBF), TBFat mass was divided by the total mass of all tissues, as follows:

PBF = (TBFat + TBLean + TBBone) × 100.

### 2.4. Blood Analyses and Indexes

Blood tests were performed at the start of the study and after 8-wks of treatment, with participants having fasted overnight for 12 h. Blood samples (10 mL) were collected into sterile tubes containing EDTA (Vacutainer^®^, Becton Dickinson Vacutainer Systems, Franklin Lakes, NJ, USA). These samples were promptly chilled on ice, and plasma was isolated through centrifugation at 1600× *g* for 10 min at 4 °C.

Laboratory analyses included: triglycerides (Tg), total cholesterol, high-density lipoprotein (HDL-c), LDL-c, alanine transaminase (ALT), aspartate aminotransferase (AST), hemoglobin, glucose, insulin, erythrocyte sedimentation rate, and monocyte, lymphocyte, neutrophil, platelet, basophil, eosinophil, red blood cell, and white blood cell counts. Blood analyses were conducted as per previously described procedures [18], at the accredited Clinical Chemical Laboratories of the University Hospital of Tor Vergata, Rome, Italy.

All assessments were conducted using the same batch of reagents or assay plates to minimize potential variability linked to variations in reagent lots.

The calculated indexes and their corresponding cut-offs are reported in Table 1.

### 2.5. RNA Extraction and RT-PCR

Blood samples were collected and preserved in PAX gene Blood RNA Tubes (Pre AnalytiX Qiagen, Hombrechtikon, Switzerland), storing them at −80 °C until RNA extraction. For each collected sample, the total RNA was purified using the PAX gene Blood miRNA Kit following the manufacturer’s provided guidelines (Pre Analytix Qiagen, Hombrechtikon, Switzerland). RNA concentration was assessed by NanoDrop ND-2000 (NanoDrop Technologies Inc., Wilmington, DE, USA). To synthesize complementary DNA (cDNA), two micrograms of total RNA samples were used. Each qRT-PCR experiment was conducted in triplicate and repeated at least twice, following the manufacturer’s instructions (Qiagen, Venlo, The Netherlands).

The following genes were amplified and analyzed: (1) *Apolipoprotein E* (*APOE*), (2) *Macrophage migration inhibitory factor* (*MIF*), (3) *SOD1*, (4) *Peroxisome Proliferator-Activated Receptor γ* (*PPARγ*), (5) *Angiotensin I-Converting Enzyme* (*ACE*), (6) *Methylenetetrahydrofolate Reductase* (*MTHFR*), (7) *CAT*, (8) *Nuclear Factor of Kappa light polypeptide gene enhancer in B-cells* (*NFkB1*), (9) *Insulin-like growth factor 2 receptor* (*IGF2R*), and (10) *USF1*. Primer sequences used for RT-PCR performance are reported in Table 2.

### 2.6. Statistical Analyses

Data collection were realized through Microsoft Office Excel^®^ (2020, Microsoft, Redmond, WA, USA).

Statistical analyses were performed by using IBM SPSS version 21.0 for Windows (IBM Corp. in Armonk, NY, USA). Following a Shapiro–Wilk test, we employed either an unpaired *t*-test or a nonparametric Wilcoxon test to assess changes before and after MedD treatment and any differences in and between the two sexes.

The differences in parameter levels (delta) values were calculated as follows:

Δ% = ((Z − W)/W) × 100, where Δ% is the percentage variation of the parameter, calculated as the ratio of absolute variation to the base value: Z is the value of the parameter after treatment, and W is the value of the parameter at baseline.

The value used to plot relative gene expression was determined using the expression Fold Change (FC) = 2^−ΔΔCT^, using B-actin (ACTB) as the housekeeping gene [26]. Raw data underwent filtration to identify genes that exhibited significant changes exceeding a factor of 1.0 within the 95% confidence interval (*p* < 0.05) for each experiment. Subsequently, only genes displaying an absolute FC value of ±2.0 or greater, along with a *p*-value < 0.05 (indicating statistical significance), were deemed as differentially expressed genes.

The results were expressed as the mean standard error of the mean (S.E.M.). The null hypothesis (no effect) was rejected at the 0.05 probability level in all statistical tests performed.

## 3. Results

### 3.1. Basic Characteristics among Females and Males

Of the thirty subjects enrolled, five did not meet the inclusion criteria, and eight did not conclude the study due to COVID-19. Therefore, seventeen participants were eligible for the study, 58.8% of whom were females and 41.2% were males. The average age of females was 28.70 ± 12.52 years, and of males was 35.57 ± 8.34 years.

From DXA analyses, before MedD treatment, significant differences were observed between females and males in gynoid total fat tissue (% and g) (*p <* 0.0001 and *p <* 0.025, respectively), total fat tissue (%) (*p <* 0.003), gynoid total fat region (%) (*p <* 0.0001), total fat region (%) (*p <* 0.003), android lean mass (*p <* 0.0001), and gynoid lean mass (*p <* 0.0001). No other significant differences were observed before MedD treatment between the two sexes. The results of the DXA parameter differences between sexes before MedD are reported in the Supplementary Materials (Table S1).

### 3.2. Effect of Intervention on Body Composition and Clinical Parameters in Females and Males

From clinical and anthropometric analyses, a significant decrease in Resistance Force (RF) (*p <* 0.009) in males after MedD treatment was observed. No significant differences in females after MedD treatment were observed. The results for the anthropometric parameter differences before and after MedD treatment in the two sexes are reported in Table 3.

Particularly, before MedD treatment, significant differences were observed between females and males in weight (*p <* 0.018), height (*p <* 0.0001), WC (*p <* 0.026), WHR (*p <* 0.0001), bicipital skinfolds (*p <* 0.008), triceps skinfolds (*p <* 0.004), RF (*p <* 0.009), and diastolic blood pressure (DBP) (*p <* 0.04). No other significant differences were observed before MedD treatment between the two sexes. After MedD treatment, significant differences were observed between females and males in weight (*p <* 0.023), WC (*p <* 0.033), WHR (*p <* 0.0001), bicipital skinfolds (*p <* 0.015), and triceps skinfolds (*p <* 0.008). No other significant differences were observed after MedD treatment between the two sexes. The results for the anthropometric parameter differences between sexes before and after MedD treatment are reported in Table 4.

From the bioimpedance analyses, no significant differences in the two sexes after MedD treatment were observed. The results for body composition differences from the bioimpedance analyses before and after MedD treatment in the two sexes are reported in Table 5.

Notably, before MedD treatment, significant differences were observed between females and males in resistance (*p <* 0.006), FFM (kg and %) (*p <* 0.0001 and *p <* 0.002, respectively), TBW (L and %) (*p <* 0.001 and *p <* 0.002, respectively), ECW (L) (*p <* 0.003), BCM (*p <* 0.001), FM (%) (*p <* 0.002), MM (kg and %) (*p <* 0.0001 and *p <* 0.013, respectively), BMR (*p <* 0.0001), and BCMI (*p <* 0.006). No other significant differences were observed before MedD treatment between sexes. After MedD treatment, significant differences were observed between females and males in resistance (*p <* 0.004), FFM (kg and %) (*p <* 0.0001 and *p <* 0.003, respectively), TBW (L and %) (*p <* 0.0001 and *p <* 0.002, respectively), ECW (L) (*p <* 0.0001), BCM (*p <* 0.0001), FM (%) (*p <* 0.003), MM (kg and %) (*p <* 0.0001 and *p <* 0.002, respectively), BMR (*p <* 0.0001), and BCMI (*p <* 0.03). No other significant differences were observed after MedD treatment between sexes. The results of body composition differences from the bioimpedance analyses between females and males before and after MedD treatment are reported in Table 6.

### 3.3. Effect of Intervention on Biochemical Parameters in Females and Males

Regarding biochemical parameters, a significant increase in hemoglobin levels (*p* < 0.04) in females was observed. No significant differences in males after MedD treatment were observed. The results of blood analyses in the two sexes before and after MedD treatment are reported in Table 7.

Particularly, before MedD treatment, significant differences were observed between females and males in hematocrit (*p <* 0.008), hemoglobin (*p <* 0.01), platelets (*p <* 0.04), and red blood cells (*p <* 0.0001). No other significant differences were observed before MedD treatment between sexes. After MedD treatment, significant differences were observed between females and males in HDL-c (*p <* 0.02), hematocrit (*p <* 0.01), hemoglobin (*p <* 0.03), platelets (*p <* 0.01), and red blood cells (*p <* 0.0001). No other significant differences were observed after MedD treatment between sexes. The results of blood analyses between females and males before and after MedD treatment are reported in Table 8.

Regarding indexes, no significant differences in the two sexes after MedD treatment were observed. The results of the indexes of the two sexes before and after MedD treatment are reported in Table 9.

Notably, before MedD treatment, significant differences were observed between females and males in PLR (*p <* 0.012). No other significant differences were observed before MedD treatment between sexes. After MedD treatment, significant differences were observed between females and males in PLR (*p <* 0.016). No other significant differences were observed after MedD treatment between sexes. The results of indexes between females and males before and after MedD treatment are reported in Table 10.

### 3.4. Effect of Intervention on Gene Expression in Females and Males

**Figure 1:**
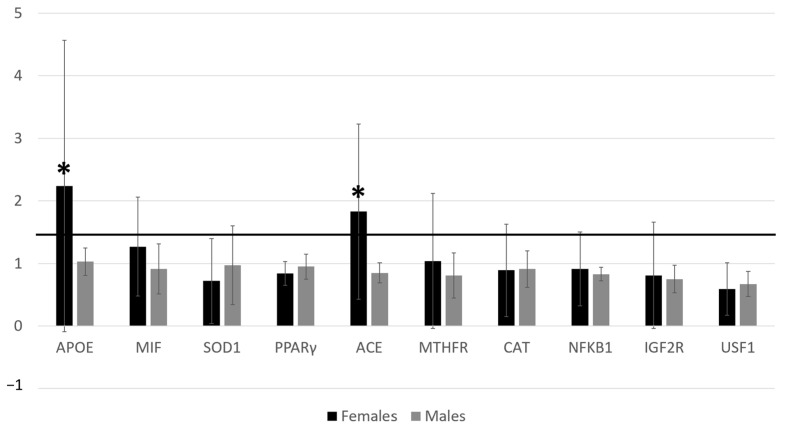
Gene expression on females and males. Black columns related to females value; grey columns related to males value. Different levels of fold changes in genes analyzed; * *p* < 0.05. Abbreviations: APOE, Apolipoprotein E; MIF, Macrophage migration Inhibitory Factor; SOD1, Superoxide Dismutase 1; PPARγ, Peroxisome Proliferator-Activated Receptor γ; ACE, Angiotensin I-Converting Enzyme; MTFHR, Methylenetetrahydrofolate Reductase; CAT, Catalase; NFkB1: Nuclear Factor of Kappa light polypeptide gene enhancer in B-cells 1; IGF2R, Insulin-like Growth Factor 2 Receptor; USF1, Upstream Transcription Factor 1.

Significant upregulation of *APOE* and *ACE*, with a fold change exceeding the threshold set at 1.5, was observed (2^−ΔΔCT^ = 2.24 and 2^−ΔΔCT^ = 1.83, respectively) in females (Figure 1). No other significant gene expression differences were observed.

## 4. Discussion

In this study, several significant differences in and between females and males were observed. Notably, a significant decrease in RF (*p* < 0.009) in males and a significant increase in hemoglobin (*p* < 0.04) in females were observed after MedD treatment. Significant differences in RF (*p <* 0.009) and DBP (*p <* 0.04) between sexes before MedD treatment were highlighted. After MedD treatment, significant differences were shown between sexes in HDL-c (*p <* 0.02). Moreover, a significant upregulation after MedD of *APOE* and *ACE* genes in females has been shown.

The MedD has been widely studied for his beneficial effects on overall health status and prevention of diseases [27]. Moreover, several studies have shown sex differences in body composition, particularly with respect to water content, lean mass, and FM and its distribution [28,29,30,31]. However, only a few studies have analyzed MedD with a focus on sex differences.

Particularly, it has been observed that young women at risk of social exclusion have lower adherence to MedD [32]. In addition, sex differences in appetite sensations have been reported in MedD treatment. Bédard et al. showed that, during and after MedD treatment, males have a significant smaller decrease in hunger, desire to eat, and appetite score, and a significant prospectively increased consumption of food before meals than females [33].

Moreover, it is well know that MedD is associated with improved health [27], enhanced well-being [34], and a positive mental state. Additionally, the MedD may reduce the risk of chronic diseases, positively impacting aging, reducing inflammation, and enhancing endothelial function and respiratory function [35]. Indeed, MedD treatment is known to improve aerobic capacity [36]. Notably, for the first time, in our study, we observed a significant difference between sexes in RF before MedD treatment, which smoothed out after treatment. This result is given by a significant decrease in RF in males after MedD treatment (*p* < 0.009). In addition, in both sexes, we observed a Δ% of Handgrip of +1.44% and +3.95%, and BCM of +2.3% and +0.7% in females and males, respectively. Schiaffino et al. [37] observed a difference in muscle fiber composition according to sex. Specifically, they observed a greater presence of white fibers, related to explosive force [38], measurable by handgrip strength, in males. In contrast, they observed a greater presence of red fibers, related to endurance [38], measurable by RF, in females. Therefore, it could be hypothesized that the decrease in RF in males could be due to an improvement in BCM, leading to an increase in white than red fibers, and thus enhancing handgrip strength against the RF.

MedD also has favorable effects on metabolic dysregulation, reducing the risk of metabolic syndrome and type 2 diabetes by acting on insulin resistance, central obesity, atherogenic dyslipidemia, and hypertension [39]. Then, for the first time, in our study, we observed a significant difference between sexes in DBP before MedD, which flattened after treatment. Notably, higher DBP values were observed in males before MedD, even if in the normal range, than in females. This may be due to several factors intrinsic to the two sexes, such as physiological mechanisms and hormonal profiles, age-related changes, and measurement techniques [40]. After MedD treatment, we did not observe significant changes in SBP and DBP in the two sexes.

We also reported, for the first time, significant differences between sexes in HDL-c after MedD treatment. Specifically, we reported an increase in HDL-c in females compared with males, while Leblanc et al., showed a better improvement in the lipid profile more in males than in females [41]. Among females, a notable impact was observed regarding MedD’s influence on various cardiovascular risk markers [34]. Specifically, Velázquez-López et al. [42] found that a high intake of dietary fiber positively affected lipid profiles, resulting in increased HDL-c levels.

A significant increase in hemoglobin after MedD was also observed in females. This could be related to fluctuations in hemoglobin values according to the phases of the menstrual cycle [43].

Furthermore, research indicates that the MedD, owing to its rich nutrients and immunonutrient profile, could influence the expression of genes associated with oxidative stress [3,4]. Indeed, several enzymes participate in the control of pathways related to oxidative stress, inflammation, and immune responses, including the *APOE* [44] and the *ACE* [45].

Specifically, the *APOE* gene encodes a protein known as *apolipoprotein E* (*APOE*), which holds a pivotal role in lipid metabolism. *APOE*’s primary function involves aiding in the absorption and removal of lipoproteins by cells. Moreover, as a component of lipoprotein particles, it can influence immune responses and inflammation through its interactions with immune cells and lipids. *APOE4* may contribute to higher levels of inflammatory markers and cytokines, which can be detrimental to tissue function and health. *APOE* isoforms have been suggested to play a role in oxidative stress due to their influence on lipid metabolism and cell interactions [44]. In our study, for the first time, we observed a significant upregulation of *APOE* in females after MedD treatment. In agreement with Liu et al. [46], who evaluated the effects of *APOE* on serum lipids in relation to sex, this could explain the significant increase we observed in HDL-c in females compared with males after MedD treatment.

Regarding *ACE*, it serves as a key regulator of blood pressure, counterbalancing the actions of vasoactive peptides within the renin–angiotensin–aldosterone system, which primarily regulates blood pressure and fluid–electrolyte balance [45]. Insertion/deletion polymorphisms in the *ACE* gene have previously been linked to an increased risk of systemic hypertension [47]. In our study, for the first time, we observed that *ACE* showed a significant upregulation in females compared to males after MedD treatment. However, we did not observe significant differences in DBP and SBP after MedD treatment in females, with values still within the normal range. This could be explained by the fact that an increase in *ACE* does not lead to hypertension unless there is a simultaneous decrease in *ACE2*, with antihypertensive effects, as reported by Pinheiro et al. [48]. Thus, a limitation of our study is that we did not analyze *ACE2*.

To the best of our knowledge, this pilot study is the first to explore the impact of the MedD on gene expression, specifically in relation to sex differences. Our findings may offer valuable insights for future research aimed at tailoring more personalized diets to meet individual needs, ultimately promoting better health homeostasis and well-being. However, it is important to acknowledge certain limitations. Firstly, due to its pilot nature, our study has a relatively small sample size, which may have also influenced the genomics results, warranting the need for larger-scale investigations. Additionally, variations among individuals aside from diet, including lifestyle, could contribute to some of the observed differences. Furthermore, our study was conducted at a single center, which may limit the generalizability of our findings to broader populations. Lastly, for a more comprehensive understanding, future research may need to explore the expression of all genes that participate in the same pathway.

## 5. Conclusions

This pilot study highlights significant sex-specific responses to the MedD. Gene expression analyses showed upregulation of *APOE* and *ACE* genes in females after MedD, indicating potential sex-specific responses to this dietary intervention. Further investigation on the interplay between diet, gene expression, and sex-specific responses, may help personalize diet and contribute to overall health and well-being.

## Figures and Tables

**Table 1:** Calculated indexes and relative cut-offs.

Index	Formula	Cut-Offs	Reference
Lipid Accumulation Product (LAP) for males	(WC [cm] − 65) × Tg concentration [mM]	20.10 to 63.89 cm mmol/L	[19]
Lipid Accumulation Product (LAP) for females	(WC [cm] − 58) × Tg concentration [mM]	25.16 to 31.59 cm mmol/L	[19]
Visceral Adiposity Index (VAI) for males	[WC/39.68 + (1.88 × BMI)] × [Tg/1.03] × 1.31/HDL-c]	2.33	[20]
Visceral Adiposity Index (VAI) for females	[WC/36.58 + (1.89 × BMI)] × [Tg/0.81] × [1.52/HDL-c]	1.32	[20]
Cardiovascular Risk Indexes	Total Cholesterol/HDL-c	≤4	[21]
	LDL-c/HDL-c	<1.5	[22]
Atherogenic Index of Plasma (AIP)	Log (Tg/HDL-c)	<0.11 low risk. ≥0.11 and ≤0.21 moderate risk. >0.21 high risk	[23]
Neutrophil/lymphocyte Ratio (NLR)	Neutrophils/Lymphocyte	<1.6 low risk. ≥1.6 and ≤2.4 moderate risk. >2.4 high risk	[24]
Platelet-to-lymphocyte Ratio (PLR)	Platelet/Lymphocyte	<150	[25]

Abbreviations: BMI, body mass index; HDL-c, high-density lipoprotein; LDL-c, low-density lipoprotein; Tg, triglycerides; WC, waist circumference.

**Table 2:** Primer sequences used for RT-PCR performance.

Target Genes	Sequences
*APO-E*	5′-GACCATGAAGGAGTTGAAGGCCTAC-3′ 3′-CTCGCGGGCCCCGGCCTGGTA-5′
*MIF*	5′-AGAACCGCTCCTACAGCAAGCT-3′ 3′-GGAGTTGTTCCAGCCCACATTG-5′
*NFκB*	5′-GAAATTCCTGATCCAGACAAAAAC-3′ 3′-ATCACTTCAATGGCCTCTGTGTAG-5′
*PPRγ*	5′-GGGATGTCTCATAATGCCATCA-3′ 3′-CGCCAACAGCTTCTCCTTCT-5′
*ACE*	5′-CTGGAGACCACTCCCATCCTTTCT-3′ 3′-GATGTGGCCATCACATTCGTCAGAT-5′
*MTHFR*	5′-TGTGGTCTCTTCATCCCTCGC-3′ 3′-CCTTTTGGTGATGCTTGTTGGC-5′
*CAT*	5′-GTGCGGAGATTCAACACTGCCA-3′ 3′-CGGCAATGTTCTCACACAGACG-5′
*SOD1*	5′-CTCACTCTCAGGAGACCATTGC-3′ 3′-CCACAAGCCAAACGACTTCCAG-5′
*IGF2-R*	5′-TTGAGTGGCGAACGCAGTATGC-3′ 3′-CAGTGATGGCTTCCCAGTTGTC-5′
*USF1*	5′-GCTCTATGGAGAGCACCAAGTC-3′ 3′-AGACAAGCGGTGGTTACTCTGC-5′

Abbreviations: APOE, Apolipoprotein E; MIF, Macrophage migration Inhibitory factor; SOD1, Superoxide Dismutase 1; PPARγ, Peroxisome Proliferator-Activated Receptor γ; ACE, Angiotensin I-Converting Enzyme; MTFHR, Methylenetetrahydrofolate Reductase; CAT, Catalase; NFkB1, Nuclear Factor of Kappa light polypeptide gene enhancer in B-cells; IGF2R, Insulin-like growth factor 2 receptor; USF1, Upstream Transcription Factor 1.

**Table 3:** Clinical and anthropometric parameter differences before and after MedD treatment in the two sexes.

	Females			Males		
	Before MedD	After MedD	*p*-Value	Before MedD	After MedD	*p*-Value
Weight (kg)	63.13 ± 9.65	62.99 ± 9.99	0.97	74.50 ± 7.02	74.12 ± 7.10	^a^ 0.71
BMI (kg/m^2^)	24.63 ± 3.87	24.57 ± 4.03	0.97	24.35 ± 1.87	24.35 ± 2.12	0.99
WC (cm)	74.23 ± 6.20	75.50 ± 5.26	0.62	83.10 ± 8.68	84.15 ± 9.91	^a^ 0.74
HC (cm)	101.69 ± 8.29	100.86 ± 8.97	0.83	96.64 ± 3.81	97.18 ± 5.24	^a^ 1.0
WHR	0.72 ± 0.02	0.74 ± 0.02	0.11	0.85 ± 0.05	0.86 ± 0.05	0.81
Bicipital Skinfold	7.73 ± 1.98	7.06 ± 2.20	0.48	4.62 ± 2.19	4.18 ± 1.97	^a^ 0.79
Triceps Skinfold	20.59 ± 5.89	20.07 ± 6.59	0.85	12.10 ± 3.25	10.47 ± 5.87	0.53
Subscapular Skinfold	14.92 ± 4.23	14.38 ± 4.16	0.77	14.19 ± 5.81	13.61 ± 5.43	^a^ 0.65
Suprailiac Skinfold	15.16 ± 4.78	14.64 ± 4.33	^a^ 0.82	10.59 ± 5.08	9.98 ± 6.09	^a^ 0.52
Handgrip Strength (kg)	23.88 ± 5.36	24.23 ± 5.42	0.89	33.12 ± 12.93	34.48 ± 12.47	0.87
RF (s)	7.18 ± 3.71	6.84 ± 2.15	0.81	16.80 ± 6.91	5.36 ± 2.09	0.009 **
SBP	107.00 ± 9.48	113.11 ± 4.96	0.1	117.29 ± 10.87	111.43 ± 6.90	0.25
DBP	71.00 ± 3.94	72.11 ± 4.19	^a^ 0.46	76.00 ± 5.80	72.14 ± 6.98	0.28

All results were expressed as mean  ±  standard deviation (SD). ^a^ Analysis was conducted using a nonparametric Wilcoxon test. Statistical significance has been attributed to results with ** *p* < 0.001. Abbreviations: BMI, body mass index; DBP, diastolic blood pressure; HC, hip circumference; MedD, Mediterranean diet; RF, resistance force; SBP, systolic blood pressure; WC, waist circumference; WHR, waist–hip ratio.

**Table 4:** Clinical and anthropometric parameters differences between females and males before and after MedD treatment.

	Before MedD	After MedD
Weight (kg)	0.018 *	0.023 *
BMI (kg/m^2^)	0.8	0.8
WC (cm)	0.026 *	0.033 *
HC (cm)	0.15	0.3
WHR	0.0001 ***	0.0001 ***
Bicipital Skinfold	0.008 **	0.015 *
Triceps Skinfold	0.004 **	0.008 **
Subscapular Skinfold	0.7	0.7
Suprailiac Skinfold	0.07	0.08
Handgrip Strength (kg)	0.1	0.051
RF (s)	0.009 **	0.2
SBP	0.056	0.5
DBP	^a^ 0.04 *	0.9

All results were expressed as mean  ±  standard deviation (SD). ^a^ Analysis was conducted using a nonparametric Wilcoxon test. Statistical significance has been attributed to results with * *p* < 0.05, ** *p* < 0.001, *** *p* < 0.0001. Abbreviations: BMI, body mass index; DBP, diastolic blood pressure; HC, hip circumference; MedD, Mediterranean diet; RF, resistance force; SBP, systolic blood pressure; WC, waist circumference; WHR, waist–hip ratio.

**Table 5:** Body composition differences from bioimpedance analyses in the two sexes before and after MedD treatment.

	Females			Males		
	Before MedD	After MedD	*p*-Value	Before MedD	After MedD	*p*-Value
Resistance (Ohm)	559.80 ± 78.30	571.16 ± 72.33	0.74	452.86 ± 46.75	462.00 ± 49.40	^a^ 0.84
Reactance (Ohm)	53.20 ± 20.83	64.25 ± 9.19	0.14	51.57 ± 4.19	54.85 ± 9.22	0.4
PhA (°)	5.99 ± 0.88	6.52 ± 0.95	0.21	6.52 ± 0.49	6.77 ± 0.81	0.51
FFM (kg)	44.83 ± 5.41	44.88 ± 5.92	0.98	62.45 ± 7.27	61.90 ± 6.33	0.88
FFM (%)	71.58 ± 6.91	71.76 ± 6.98	0.95	83.84 ± 6.42	83.60 ± 6.10	0.94
TBW (L)	33.07 ± 3.70	33.09 ± 4.03	0.99	45.70 ± 5.31	45.31 ± 4.64	0.88
TBW (%)	52.80 ± 4.46	52.92 ± 4.33	0.95	61.37 ± 4.70	61.20 ± 4.48	0.94
ECW (L)	15.58 ± 2.49	14.42 ± 1.73	0.24	19.85 ± 2.29	19.34 ± 2.67	0.7
ECW (%)	46.00 ± 4.16	43.75 ± 4.19	0.24	43.47 ± 2.21	42.60 ± 3.28	0.57
BCM (kg)	24.65 ± 5.09	25.22 ± 4.57	0.79	35.10 ± 4.61	35.34 ± 3.88	0.91
FM (kg)	18.30 ± 6.59	18.11 ± 6.26	0.94	12.04 ± 4.89	12.22 ± 4.97	0.94
FM (%)	28.42 ± 6.91	28.24 ± 6.98	0.95	16.15 ± 6.42	16.40 ± 6.10	0.94
Na/K	1.00 ± 0.13	0.94 ± 0.11	^a^ 0.32	1.08 ± 0.12	1.04 ± 0.15	0.57
ICW (%)	54.00 ± 4.16	56.25 ± 4.19	0.24	56.52 ± 2.21	57.40 ± 3.28	0.57
MM (kg)	29.57 ± 4.59	30.34 ± 6.25	0.75	41.47 ± 6.32	43.07 ± 4.59	0.59
MM (%)	47.20 ± 5.94	49.22 ± 6.53	0.47	55.62 ± 6.26	59.64 ± 3.75	0.17
BMR	1447.02 ± 115.5	1481.09 ± 132.37	0.54	1768.31 ± 134.27	1774.98 ± 112.23	0.92
BCMI	9.36 ± 1.46	9.82 ± 1.70	0.52	11.48 ± 1.14	11.64 ± 1.24	0.81

All results were expressed as mean  ±  standard deviation (SD). ^a^ Analysis was conducted using a nonparametric Wilcoxon test. Abbreviations: BCM, body cell mass; BCMI, body cell mass index; BMR, metabolic resting rate; ECW, extra-cellular water; FFM, fat-free mass; FM, fat mass; ICW, intra-cellular water; MedD, Mediterranean diet; MM, muscle mass; Na/K, sodium–potassium ratio; PhA, phase angle; TBW, total body water.

**Table 6:** Body composition differences from bioimpedance analyses between females and males before and after MedD treatment.

	Before MedD	After MedD
Resistance (Ohm)	0.006 **	0.004 **
Reactance (Ohm)	0.8	0.056
PhA (°)	0.1	0.5
FFM (kg)	0.0001 ***	0.0001 ***
FFM (%)	0.002 **	0.003 **
TBW (L)	^a^ 0.001 **	0.0001 ***
TBW (%)	0.002 **	0.002 **
ECW (L)	0.003 **	0.0001 **
ECW (%)	0.1	0.5
BCM (kg)	0.001 **	0.0001 ***
FM (kg)	0.051	0.057
FM (%)	0.002 **	0.003 **
Na/K	0.1	^a^ 0.1
ICW (%)	0.1	0.5
MM (kg)	0.0001 ***	0.0001 ***
MM (%)	0.013 **	0.002 **
BMR	0.0001 ***	0.0001 ***
BCMI	0.006 **	0.03 *

All results were expressed as mean ± standard deviation (SD). ^a^ Analysis was conducted using a nonparametric Wilcoxon test. Statistical significance has been attributed to results with * *p* < 0.05, ** *p* < 0.001, *** *p* < 0.0001. Abbreviations: BCM, body cell mass; BCMI, body cell mass index; BMR, metabolic resting rate; ECW, extra-cellular water; FFM, fat-free mass; FM, fat mass; ICW, intra-cellular water; MedD, Mediterranean diet; MM, muscle mass; Na/K, sodium–potassium ratio; PhA, phase angle; TBW, total body water.

**Table 7:** Blood tests differences in the two sexes before and after MedD treatment.

	Females			Males		
	Before MedD	After MedD	*p*-Value	Before MedD	After MedD	*p*-Value
Glycemia (mg/dL)	80.40 ± 7.58	76.20 ± 8.91	0.27	83.86 ± 8.72	79.71 ± 10.53	0.43
Insulin (μU/mL)	6.90 ± 3.06	6.93 ± 2.84	0.98	7.58 ± 6.07	7.20 ± 4.80	0.89
Total Cholesterol (mg/dL)	165.60 ± 17.04	174.50 ± 26.11	0.37	159.14 ± 18.56	163.43 ± 18.36	0.67
HDL-c (mg/dL)	65.00 ± 8.85	67.10 ± 8.31	0.59	58.14 ± 10.97	57.57 ± 5.85	0.9
Tg (mg/dL)	65.90 ± 31.01	62.40 ± 21.94	0.77	77.86 ± 60.34	72.43 ± 57.66	0.86
LDL-c (mg/dL)	90.70 ± 12.30	100.50 ± 16.57	0.15	93.14 ± 10.18	96.57 ± 16.04	0.64
AST (U/L)	19.40 ± 16.06	22.00 ± 18.73	^a^ 0.73	30.86 ± 25.19	17.86 ± 5.36	^a^ 0.56
ALT (U/L)	23.10 ± 7.21	25.30 ± 13.01	^a^ 0.82	28.86 ± 6.66	24.00 ± 5.91	0.17
Basophils (cells/mL)	0.02 ± 0.01	0.02 ± 0.01	0.65	0.02 ± 0.01	0.02 ± 0.01	^a^ 0.72
Basophils (%)	0.43 ± 0.33	0.30 ± 0.13	^a^ 0.42	0.45 ± 0.30	0.37 ± 0.12	0.5
Eosinophils (cells/mL)	0.10 ± 0.09	0.12 ± 0.09	^a^ 0.51	0.15 ± 0.11	0.15 ± 0.06	0.97
Hematocrit (%)	38.96 ± 2.58	39.94 ± 1.86	0.34	43.01 ± 2.87	42.87 ± 2.75	0.92
Hb (g/dL)	13.12 ± 0.96	13.40 ± 0.67	^a^ 0.04 *	14.77 ± 1.37	14.61 ± 1.36	^a^ 0.69
Lymphocytes (cells/mL)	1.99 ± 0.49	2.06 ± 0.52	^a^ 0.88	2.01 ± 0.36	2.07 ± 0.24	0.72
Lymphocytes (%)	31.51 ± 6.58	28.37 ± 7.24	0.32	32.58 ± 4.67	35.00 ± 7.35	0.47
MCH (pg)	30.75 ± 1.76	30.64 ± 1.93	0.89	29.75 ± 3.13	29.48 ± 3.15	0.87
MCHC (g/dL)	33.66 ± 0.92	33.67 ± 0.97	0.98	34.28 ± 1.34	34.05 ± 1.30	^a^ 0.52
MCV (Fl)	91.38 ± 4.47	91.03 ± 4.50	0.86	86.64 ± 6.94	86.51 ± 7.05	^a^ 0.84
Monocytes (cells/mL)	0.41 ± 0.08	0.45 ± 0.11	0.39	0.43 ± 0.08	0.41 ± 0.13	0.66
Monocytes (%)	6.66 ± 1.46	6.26 ± 1.37	0.53	7.05 ± 1.21	6.58 ± 1.43	0.51
Neutrophils (cells/mL)	3.82 ± 0.97	4.82 ± 1.44	0.08	3.60 ± 0.85	3.61 ± 1.91	^a^ 0.09
Neutrophils (%)	59.63 ± 7.84	63.43 ± 7.63	0.28	57.54 ± 5.27	55.54 ± 8.25	0.59
Platelets (cells/mL)	270.90 ± 67.32	285.10 ± 65.81	0.63	210.43 ± 32.70	213.14 ± 32.59	0.87
Red Blood Cells (million/μL)	4.27 ± 0.35	4.39 ± 0.28	0.4	4.97 ± 0.16	4.96 ± 0.20	0.94
RDW-CV (%)	13.04 ± 0.58	13.19 ± 0.66	^a^ 0.62	13.17 ± 0.70	13.10 ± 0.78	0.86
White Blood Cells (cells/mL)	6.36 ± 1.06	7.50 ± 1.70	0.09	6.24 ± 1.13	6.27 ± 2.11	^a^ 0.48

All results were expressed as mean ± standard deviation (SD). ^a^ Analysis was conducted using a nonparametric Wilcoxon test. Statistical significance has been attributed to results with * *p* < 0.05. Abbreviations: ALT, alanine transaminase; AST, aspartate aminotransferase; Hb, hemoglobin; HDL-c, high-density lipoprotein; MCH, mean corpuscular hemoglobin; MCHC, mean corpuscular hemoglobin concentration; MCV, mean cell volume; LDL-c, low-density lipoprotein; RDW-CV, red cell distribution width; Tg, triglycerides.

**Table 8:** Blood tests differences between females and males before and after MedD treatment.

	Before MedD	After MedD
Glycemia (mg/dL)	^a^ 0.4	^a^ 0.4
Insulin (μU/mL)	0.7	0.8
Total Cholesterol (mg/dL)	0.4	0.3
HDL-c (mg/dL)	0.1	0.02 *
Tg (mg/dL)	^a^ 0.9	0.6
LDL-c (mg/dL)	0.6	0.6
AST (U/L)	0.2	^a^ 0.7
ALT (U/L)	0.1	^a^ 0.6
Basophils (cells/mL)	0.8	0.8
Basophils (%)	0.8	0.2
Eosinophils (cells/mL)	0.3	0.4
Hematocrit (%)	0.008 **	0.01 *
Hb (g/dL)	0.01 *	0.03 *
Lymphocytes (cells/mL)	0.9	^a^ 0.4
Lymphocytes (%)	0.7	0.08
MCH (pg)	0.4	0.3
MCHC (g/dL)	0.2	0.4
MCV (Fl)	0.1	0.1
Monocytes (cells/mL)	^a^ 0.8	0.4
Monocytes (%)	0.5	0.6
Neutrophils (cells/mL)	0.6	0.1
Neutrophils (%)	0.5	0.06
Platelets (cells/mL)	0.04 *	0.01 *
Red Blood Cells (million/μL)	0.0001 ***	0.0001 ***
RDW-CV (%)	0.6	^a^ 0.7
White Blood Cells (cells/mL)	0.8	0.2

All results were expressed as mean ± standard deviation (SD). ^a^ Analysis was conducted using a nonparametric Wilcoxon test. Statistical significance has been attributed to results with * *p* < 0.05, ** *p* < 0.001, *** *p* < 0.0001. Abbreviations: ALT, alanine transaminase; AST, aspartate aminotransferase; Hb, hemoglobin; HDL-c, high-density lipoprotein; MCH, mean corpuscular hemoglobin; MCHC, mean corpuscular hemoglobin concentration; MCV, mean cell volume; LDL-c, low-density lipoprotein; RDW-CV, red cell distribution width; Tg, triglycerides.

**Table 9:** Index differences in the two sexes before and after MedD treatment.

	Females			Males		
	Before MedD	After MedD	*p*-Value	Before MedD	After MedD	*p*-Value
LAP	10.64 ± 2.60	11.80 ± 4.22	0.47	19.32 ± 25.55	19.22 ± 26.49	^a^ 0.84
VAI	0.74 ± 0.37	0.70 ± 0.27	^a^ 0.94	0.80 ± 0.70	0.73 ± 0.65	0.84
Total Cholesterol/HDL-c	2.56 ± 0.23	2.60 ± 0.27	0.73	2.80 ± 0.51	2.87 ± 0.52	^a^ 0.84
LDL-c/HDL-c	1.40 ± 0.21	1.50 ± 0.24	0.34	1.66 ± 0.43	1.70 ± 0.39	0.86
AIP	−0.026 ± 0.18	−0.056 ± 0.18	0.71	0.047 ± 0.33	0.01 ± 0.31	0.83
NLR	2.01 ± 0.67	2.42 ± 0.84	0.24	1.81 ± 0.41	1.74 ± 0.87	^a^ 0.27
PLR	137.66 ± 25.84	141.28 ± 32.80	0.78	105.92 ± 16.76	103.71 ± 19.08	0.82

All results were expressed as mean ± standard deviation (SD). ^a^ Analysis was conducted using a nonparametric Wilcoxon test. Abbreviations: AIP, atherogenic index of plasma; HDL-c, high-density lipoprotein; LAP, lipid accumulation product; LDL-c, low-density lipoprotein; NLR, neutrophil/lymphocyte ratio; PLR, platelet-to-lymphocyte ratio; VAI, visceral adiposity index.

**Table 10:** Index differences between females and males before and after MedD treatment.

	Before MedD	After MedD
LAP	^a^ 0.8	^a^ 0.5
VAI	^a^ 0.3	^a^ 0.4
Total Cholesterol/HDL-c	0.2	0.1
LDL-c/HDL-c	0.1	0.2
AIP	0.5	0.5
NLR	0.4	^a^ 0.1
PLR	0.012 *	0.016 *

All results were expressed as mean ± standard deviation (SD). ^a^ Analysis was conducted using a nonparametric Wilcoxon test. Statistical significance attributed to results with * *p* < 0.05. Abbreviations: AIP, atherogenic index of plasma; HDL-c, high-density lipoprotein; LAP, lipid accumulation product; LDL-c, low-density lipoprotein; NLR, neutrophil/lymphocyte ratio; PLR, platelet-to-lymphocyte ratio; VAI, visceral adiposity index.

## Data Availability

This published article includes all the data generated or analyzed during this study.

## Supplementary Materials

The following supporting information can be downloaded at: https://www.mdpi.com/article/10.3390/genes14111980/s1, Table S1: DXA parameter differences between females and males before MedD treatment.
