# Supplementary material for: Sex Differences in the Efficacy of Mediterranean Diet Treatment: A Nutrigenomics Pilot Study
Source: Genes (Basel). 2023 Oct 24;14(11):1980. doi: 10.3390/genes14111980 (PMC10671063; doi:10.3390/genes14111980)
Supplement: Supplementary file 1 [file genes-14-01980-s001.zip › genes-2667125-supplementary.pdf]

genes

Table S1. DXA parameter differences between females and males before MedD treatment.

	Females	Males	*p*-value
Android Total Fat Tissue (%)	33.90 ± 9.97	22.71 ± 14.58	0.08
Android Total Fat Tissue (g)	1425.10 ± 750.07	1253.67 ± 1110.65	0.7
Gynoid Total Fat Tissue (%)	40.57 ± 6.18	22.78 ± 8.14	0.0001***
Gynoid Total Fat Tissue (g)	4064.10 ± 1290.56	2483.83 ± 1067.39	0.025*
Total Fat Tissue (%)	35.27 ± 6.44	21.51 ± 8.60	0.003**
Total Fat Tissue (g)	18201.70 ± 6776.74	15841.83 ± 7753.28	0.53
Android Total Fat Region (%)	32.64 ± 10.11	22.51 ± 14.49	0.1
Gynoid Total Fat Region (%)	39.73 ± 6.17	22.20 ± 8.01	0.0001***
Total Fat Region (%)	33.99 ± 6.33	20.70 ± 8.33	0.003**
Android Tissue (g)	3962.30 ± 1006.95	4959.00 ± 1178.54	0.9
Gynoid Tissue (g)	9885.70 ± 1831.26	8877.33 ± 4394.32	0.52
Total Tissue (g)	100934.00 ± 125044.32	71979.50 ± 7376.00	0.58
Android Lean Mass (g)	2537.20 ± 368.50	3705.67 ± 413.54	0.0001***
Gynoid Lean Mass (g)	5794.70 ± 839.92	8238.17 ± 704.88	0.0001***
Total Lean Mass (g)	66330.50 ± 85698.90	47299.83 ± 23441.10	0.6

All results were expressed as mean ± standard deviation (SD). Statistical significance has been attributed to results with $*p < 0.05$, $**p < 0.001$, $***p < 0.0001$.
